# Bioinformatics approaches for studying molecular sex differences in complex diseases

**DOI:** 10.1093/bib/bbae499

**Published:** 2024-10-14

**Authors:** Rebecca Ting Jiin Loo, Mohamed Soudy, Francesco Nasta, Mirco Macchi, Enrico Glaab

**Affiliations:** Biomedical Data Science Group, Luxembourg Centre for Systems Biomedicine (LCSB), University of Luxembourg, 6 avenue du Swing, L-4367 Belvaux, Luxembourg; Biomedical Data Science Group, Luxembourg Centre for Systems Biomedicine (LCSB), University of Luxembourg, 6 avenue du Swing, L-4367 Belvaux, Luxembourg; Biomedical Data Science Group, Luxembourg Centre for Systems Biomedicine (LCSB), University of Luxembourg, 6 avenue du Swing, L-4367 Belvaux, Luxembourg; Biomedical Data Science Group, Luxembourg Centre for Systems Biomedicine (LCSB), University of Luxembourg, 6 avenue du Swing, L-4367 Belvaux, Luxembourg; Biomedical Data Science Group, Luxembourg Centre for Systems Biomedicine (LCSB), University of Luxembourg, 6 avenue du Swing, L-4367 Belvaux, Luxembourg

**Keywords:** molecular sex differences, complex diseases, pathway and network analysis, bioinformatics, biomarker signature modeling, personalized medicine

## Abstract

Many complex diseases exhibit pronounced sex differences that can affect both the initial risk of developing the disease, as well as clinical disease symptoms, molecular manifestations, disease progression, and the risk of developing comorbidities. Despite this, computational studies of molecular data for complex diseases often treat sex as a confounding variable, aiming to filter out sex-specific effects rather than attempting to interpret them. A more systematic, in-depth exploration of sex-specific disease mechanisms could significantly improve our understanding of pathological and protective processes with sex-dependent profiles. This survey discusses dedicated bioinformatics approaches for the study of molecular sex differences in complex diseases. It highlights that, beyond classical statistical methods, approaches are needed that integrate prior knowledge of relevant hormone signaling interactions, gene regulatory networks, and sex linkage of genes to provide a mechanistic interpretation of sex-dependent alterations in disease. The review examines and compares the advantages, pitfalls and limitations of various conventional statistical and systems-level mechanistic analyses for this purpose, including tailored pathway and network analysis techniques. Overall, this survey highlights the potential of specialized bioinformatics techniques to systematically investigate molecular sex differences in complex diseases, to inform biomarker signature modeling, and to guide more personalized treatment approaches.

## Introduction

Many complex diseases show pronounced differences between the sexes that may influence not only the clinical manifestations and molecular phenotypes, but also the risk of disease development *a priori*, response to specific therapies, and long-term disease progression and outcome. Sex differences have been reported in all major categories of common human disorders, including cardiovascular diseases [1, 2], cancers [3, 4], neurological disorders [5, 6], metabolic disorders [7, 8], rheumatoid diseases [9, 10], autoimmune diseases [11, 12], pulmonary diseases [13, 14], musculoskeletal disorders [15, 16], infectious diseases [17, 18], and genetic disorders [19, 20].

The disparities between the sexes in these disorders are often not limited to a single symptom or molecular manifestation of disease, but exhibit a multifaceted pattern that influences the entire spectrum of disease presentation, including long-term progression and prognosis. Even within the same disease, sex-specific patterns can vary significantly across different symptoms and disease characteristics. For example, the autoimmune disease multiple sclerosis affects men and women differently in many aspects, with women often experiencing more severe fatigue [21, 22], while males tend to have a faster progression of disability and more destructive lesions [23]. Moreover, the profile of sex differences is often disease-specific, and even related disorders, such as neurodegenerative disorders, can have very different sex-specific risk profiles and manifestations. For instance, a higher age-adjusted prevalence of Alzheimer’s disease (AD) is observed in women than in men [24, 25], but an opposite pattern has been reported for Parkinson’s disease (PD) [26, 27]. In addition, in AD, women have more extensive pathology [28], while men experience a more rapid disease progression and earlier mortality [29], and in PD, women are more likely to have resting tremor as a primary symptom, but this is associated with less severe motor decline and striatal degeneration [30]. To add to the complexity, within the same disease, different genetic subtypes may show distinct sex-specific patterns, as seen in PD, where familial forms of the disease with a mutation in the gene *LRRK2* show an increased female prevalence [31, 32], in contrast to the increased male prevalence observed in idiopathic PD.

For most of these sex-specific features of complex diseases, the underlying causes and mediating molecular factors and mechanisms remain unknown or incompletely understood. This lack of comprehensive understanding underscores the need for scientific research on disease-related sex differences, as it holds the potential to reveal relevant disease-modifying biological mechanisms and may have practical implications for personalized treatment strategies and public health interventions.

Traditionally, computational studies of molecular data for complex diseases have often treated sex as a confounding factor. The focus has been on filtering out sex-specific effects rather than interpreting and understanding them. While this approach is methodologically sound and appropriate for certain types of analyses, it potentially overlooks critical insights into the sex-specific mechanisms that underlie these diseases. A more systematic and comprehensive exploration of these sex-specific disease mechanisms could significantly enhance our understanding of both pathological and protective processes that have sex-specific profiles. Such a systematic and mechanism-guided investigation can exploit the synergies of multiple sources of prior information by using dedicated bioinformatics approaches to integrate molecular datasets with existing mechanistic knowledge. Public molecular interaction and functional annotation databases provide detailed data on the regulation and interaction of sex hormones and steroids, on sex-linked genes, and the upstream and downstream molecular signaling chains in which these genes are involved. While this information is not always available in tissue- and cell-type specific forms, dedicated bioinformatics approaches can help to contextualize the available data for the disease-associated cell types of interest [33, 34]. Beyond corroborating statistical evidence of disease-related sex differences for individual genes or biomolecules with prior mechanistic knowledge, bioinformatics approaches can also reveal broader patterns of sex differences in molecular activity at the level of coordinated changes in cellular pathways and sub-networks. In addition, dedicated bioinformatics algorithms have the potential to simulate the effects of targeted molecular network perturbations and to reveal key upstream regulators of sex-differential pathways, thereby providing valuable insights for potential therapeutic interventions.

To help address the limited consideration of sex differences in many molecular studies of complex diseases, this survey uniquely integrates a comprehensive range of analytical methods for studying sex differences in complex diseases, from basic statistical approaches to advanced network analyses. By providing a first high-level overview of the analytical landscape, we offer researchers a roadmap for selecting and applying appropriate methods. We discuss how prior knowledge of hormone signaling pathways and regulatory interactions of sex-linked genes can be exploited, pointing out the advantages of different bioinformatics approaches and data sources, but also addressing common pitfalls and limitations. The manuscript progresses from advice on biostatistical evaluation of different types of molecular sex differences and identification of appropriate sources of prior data to a comparison of approaches for mechanistic analysis of sex-dependent changes in cellular processes and their key upstream regulators. Our integrative perspective highlights new opportunities to uncover sex-specific disease mechanisms that may be missed by more narrowly focused studies. This approach can be instrumental in advancing personalized medicine by providing a more nuanced understanding of how sex influences disease risk, progression, and response to treatment. Findings from these analyses can directly inform the development of tailored diagnostic tools and targeted interventions, potentially revolutionizing clinical practice. In addition, these insights can guide the formulation of sex-specific public health policies, from targeted screening programs to population-level prevention strategies, ultimately contributing to more effective and equitable healthcare systems.

**Figure 1:**
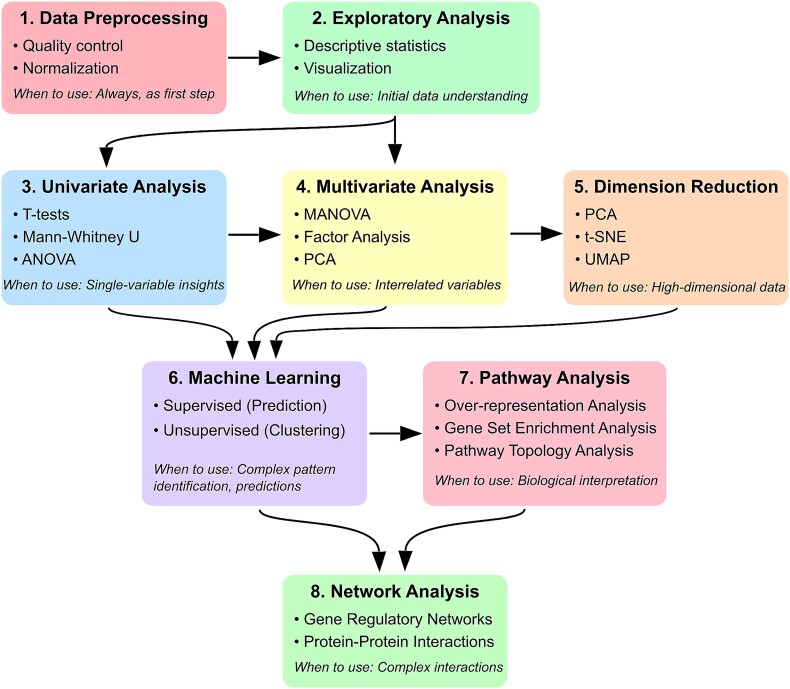
Roadmap of analysis methods for sex differences in complex diseases. This flowchart outlines a comprehensive approach to analyzing sex differences, progressing from basic data preprocessing to advanced network analysis. Each main category (in colored boxes) represents a distinct class of analytical tools, with sub-categories and specific methods listed below. The hierarchy from top to bottom reflects increasing incorporation of prior biological knowledge and indicates increasing algorithmic complexity. Not every methodology may be required for each application, and the text at the bottom of the boxes indicates when to use each approach. Steps can be iterative and may not always follow a linear progression. While the focus is on interpreting sex differences, these methods are applicable to investigate both sex-dependent and sex-neutral disease-associated changes.

To provide an overview of the analytical approaches discussed in this review, Fig. 1 presents a roadmap for applying common methods used to investigate both sex-dependent and sex-neutral changes in complex diseases. This figure illustrates the progression from basic data preprocessing and statistical techniques to more advanced computational and systems biology approaches. The methods are grouped into eight main categories: Data Preprocessing, Exploratory Analysis, Univariate Analysis, Multivariate Analysis, Dimension Reduction, Machine Learning, Pathway Analysis, and Network Analysis. Each category is further divided into subcategories or examples for specific techniques, reflecting the increasing complexity and biological context incorporated into the analyses. This overview of methods not only serves as a roadmap for the subsequent sections of this review, but also highlights the interdisciplinary nature of studying complex diseases, with a particular emphasis on sex differences. As the review progresses, we will discuss each of the main methodological approaches in detail, exploring their applications, advantages, and limitations in the context of both sex-dependent and sex-neutral disease manifestations and progression.

While we do not provide new empirical validations for specific methodologies within the scope of this study, we extensively reference previous results from the literature that provide empirical support. These referenced studies often include functional assays and experimental validations that confirm the role of specific genes or pathways in sex differences. By compiling and analyzing these empirically validated results, we aim to provide a robust overview of the current state of knowledge and methodological approaches in the field. Readers interested in the detailed empirical validation of specific methodologies or findings are encouraged to refer to the original research articles cited throughout this review.

Overall, the motivation for this survey lies in the recognition that molecular sex differences in complex diseases represent a rich and often relatively untapped source of information. Unlocking this information through dedicated bioinformatics approaches has the potential to significantly improve our understanding of molecular mechanisms in complex diseases, and guide research towards more effective and personalized therapies.

## Categorization of sex differences in complex diseases

### Categorization of sex differences by disease stages

**Figure 2:**
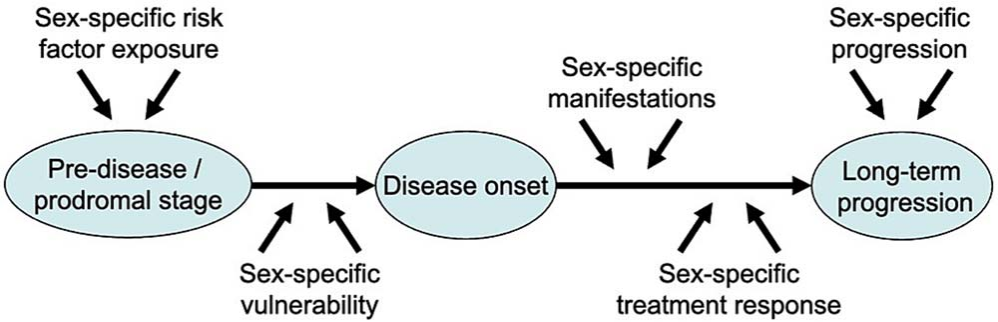
Categorization of disease-associated sex differences according to disease stage. Sex differences can affect an individual’s exposure to risk and protective factors and vulnerability to toxic insults prior to disease onset, as well as the manifestation, response to treatment, and long-term course of a disease.

Sex differences can influence every stage of disease development and progression, requiring a thorough understanding of how environmental, endogenous molecular, and genetic factors may differentially affect disease risk, onset, and outcomes in women and men. We propose a conceptual framework for categorizing disease-associated sex differences by disease stages, as illustrated in Fig. 2. This framework accounts for the continuum of the disease process, including differential vulnerability and risk exposure prior to disease development, and categorizes it into three primary stages: Prodromal/pre-disease stage, disease onset, and long-term disease progression. Each of these stages can be influenced by sex-specific factors, such as risk and protective factor exposures, susceptibilities, manifestations, treatment responses, and progression patterns. Understanding the specific influences of sex at different stages for a disease of interest may be relevant both for the development of diagnostic and prognostic methods and for the design of new therapies for sex-dependent pathologies.

#### Prodromal / pre-disease stage

This initial stage focuses on sex-specific risk exposures that may predispose individuals to certain diseases. For example, men and women experience different hormonal influences, such as protective effects of higher estrogen levels in women, which can modify the risk of cardiovascular disease and osteoporosis. Lifestyle factors may also play a role, as potential sex differences in behaviors, such as smoking and diet, may contribute to the risk of diseases such as lung cancer and heart disease.

### Disease onset stage

This stage reflects the transition from risk factors to the actual development of the disease. Sex-specific susceptibilities, such as genetic predispositions (e.g. mutations in the *BRCA1* gene, which are associated with a higher risk of breast cancer in women [35]), can influence the likelihood of disease onset. In addition, some autoimmune diseases, such as systemic lupus erythematosus, are more common in women, possibly due to sex-related immunological or hormonal factors [36].

#### Long-term progression stage

At this stage, the differences in how diseases manifest and progress in men and women become apparent. Chronic diseases may progress differently; e.g. women may experience more severe progression of autoimmune diseases [37, 38], while men may experience more aggressive motor symptom progression of Parkinson’s disease [39, 40]. Treatment responses may also differ by sex, with some drugs having different efficacy or side effect profiles in men and women.

In summary, the impact of sex on complex disorders can be highly stage-specific. By systematically considering sex differences in disease risk, manifestation, and progression for a disorder of interest in the context of this stage-specific conceptual framework, researchers and health care professionals may have the potential to better predict, diagnose, and manage diseases and associated comorbidities and symptoms for both sexes.

### Categorization of disease-associated molecular changes by their specificity and directionality across the sexes

**Figure 3:**
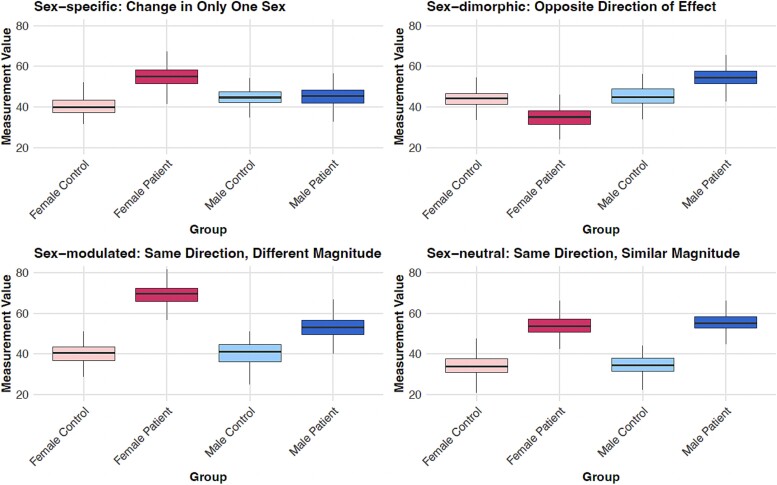
Representative illustration of different categories of sex dependencies in disease-associated molecular abundance changes (using simulated data). Top left: Sex-specific changes, i.e. changes that are significant only in one sex and do not approach significance in the other sex; top right: Sex-dimorphic changes, i.e. significant changes in both sexes with opposite direction of effect between the sexes; lower left: Sex-modulated changes, i.e. significant changes in both sexes with the same direction of the effect, but a significant difference in the magnitude of the effect; bottom right: Sex-neutral changes, i.e. significant changes in both sexes with the same direction and similar magnitude of effect.

Beyond categorizing disease-associated sex differences by stage, the specific sex-dependent molecular changes within a disorder can be systematically grouped by both their directionality and sex specificity. As part of the conceptual framework proposed here, we group individual disease-associated molecular changes into four classes:

1) Sex-specific changes: Sex-specific alterations are molecular differences between patients and controls that are detected in only one of the sexes (for practical statistical analyses, they can be defined as alterations that are significant in only one sex, e.g. with a false discovery rate (FDR) p-value <0.05, and that do not approach significance in the other sex - a conservative threshold for non-significance in the other sex should be chosen to avoid spurious specificity assignments, e.g. a nominal p-value >0.5). Corresponding patterns may reflect a disease mechanism or biological response to a disease that is unique to one sex, or that is frequent and pronounced in only one sex. An example of a typical box plot representation of a sex-specific change is shown in Fig. 3, top left.
2) Sex-dimorphic changes: Sex-dimorphic changes are detected as statistically significant in both sexes (FDR < 0.05), but diverge in the direction of their log fold change (logFC; for robustness, a minimum logFC difference of 0.5 can be defined as an additional criterion). Such changes may e.g. reflect distinct regulatory mechanisms triggered by the same disease stimuli in men and women. An example box plot for a sex-dimorphic change is shown in Fig. 3, top right.
3) Sex-modulated changes: Sex-modulated changes are significant in both sexes (FDR < 0.05) with the same direction of change but significantly different in magnitude (see example box plot in Fig. 3, bottom left). Such patterns may reflect an overall response to the disease that is the same in males and females, but with a severity or magnitude of response that is sex specific.
4) Sex-neutral changes: Finally, sex-neutral changes are molecular differences that are significant in both sexes (FDR < 0.05), have the same direction of change, and show no significant difference in the magnitude of change between the sexes (see example in Fig. 3, bottom right). These changes reflect shared pathways and mechanisms in disease pathology between the sexes.

#### Categorization of sex differences by their origin and the analysis types required

In addition to the above categorizations of sex differences by disease stage and type of changes observed, a general conceptual guide to the statistical analysis of sex differences in both animal models and humans by Beltz et al. proposed a subdivision by the origin of sex differences and associated types of analyses [41]. Emphasizing the importance of recognizing sex as a biological variable (SABV) in the design and analysis of biomedical studies in general, their work categorizes sex differences into qualitative, quantitative, latent, and population differences, each of which requires specific analysis strategies. Key conclusions are that qualitative differences require separate analyses for each sex, while quantitative differences require the use of sex as a factor in the analysis. In addition, it is noted that latent and population sex differences may occur when there are differences in the mechanisms underlying a trait or in the proportions of women and men exhibiting certain traits in response to an independent variable. In these cases, the choice of the appropriate methodology will depend on the trait or condition being studied, and the authors provide specific examples of how to deal with different scenarios [42].

To provide a structured overview of the different ways sex differences can be categorized in complex diseases, Table 1 presents a summary of the main categorization approaches discussed here.

**Table 1:** Categorizations of sex differences in complex diseases. This table summarizes three main approaches to categorizing sex differences in complex diseases: By disease stage, by molecular change pattern, and by origin and analysis type. Each approach is further divided into specific categories, providing a comprehensive framework for understanding and analyzing sex differences across various aspects of disease manifestation and progression.

Categorization Approach	Categories	Description
By Disease Stage	1. Prodromal/pre-disease stage	Sex differences in risk factors, exposures, and susceptibilities
	2. Disease onset stage	Sex-specific susceptibilities and initial manifestations
	3. Long-term progression stage	Sex differences in disease progression, symptoms, and treatment responses
By Molecular Change Pattern	1. Sex-specific changes	Molecular alterations detected only in one sex
	2. Sex-dimorphic changes	Significant changes in both sexes with opposite directions of the change
	3. Sex-modulated changes	Significant changes in both sexes with same direction but different magnitudes
	4. Sex-neutral changes	Significant changes in both sexes with same direction and similar magnitudes
By Origin and Analysis Type (Betz et al., 2019)	1. Qualitative differences	Require separate analyses for each sex
	2. Quantitative differences	Require using sex as a factor in the analysis
	3. Latent differences	Differences in mechanisms underlying a trait
	4. Population differences	Differences in proportions of traits in response to an independent variable

## Univariate statistical analysis of sex differences in complex diseases

When assigning specific molecular changes measured for a disease state to the categories of sex-specific, sex-dimorphic, sex-modulated, and sex-neutral, researchers must be aware of the pitfalls of associated statistical analyses, including the limitations of sex-stratified analyses and the challenges of analyzing interactions between sex and disease state. Sex-stratified analyses use standard statistical tests for differential molecular abundance analysis to test for disease-associated changes in each sex separately. These may include classical parametric hypothesis tests, such as Welch’s test for normally distributed data, or non-parametric tests, such as the Mann–Whitney U test, as well as special moderated statistics for high-dimensional omics data analysis, such as the empirical Bayes moderated t-statistic [43].

However, a pure sex-stratified analysis may misclassify a change as sex-specific if it uses a standard significance threshold to assess both the presence and absence of an effect. Stochastic variation in significance scores around a chosen threshold may lead to the erroneous detection of significance specific to only one sex, especially if the p-value in the other sex marginally exceeds the chosen threshold. In addition, such an analysis may miss sex-modulated changes, where significant changes in both sexes share the same direction but differ significantly in magnitude, these changes require cross-sex comparisons for accurate detection.

In contrast, analyses that examine interactions between sex and disease status, while theoretically adept at detecting sex-modulated changes, present different challenges. These approaches typically use linear models that include an interaction term for sex and disease status (i.e. the term ‘sex*disease,’ where sex and disease are factors in the model) to determine whether the relationship between disease and molecular changes differs significantly between males and females. Not only can such interaction terms reveal complexities in disease mechanisms that might otherwise be obscured in analyses that do not consider SABV, but they also have the potential to detect changes that are limited to the magnitude of an effect, an aspect that sex-stratified analyses do not capture. Nevertheless, robust estimation of interaction effects requires large sample sizes, which are often not available due to the costs associated with advanced molecular profiling techniques such as single-cell RNA sequencing.

An alternative approach to using an interaction term analysis is to combine sex-stratified analyses with a so-called ‘differential difference’ or ‘contrast of contrasts’ analysis that compares the difference between female cases and controls with the difference between male cases and controls (e.g. using a linear model formulation with a term such as ‘(female_case – female_control) – (male_case – male_control)’). While this method provides easily interpretable information about the difference in disease effects between the sexes and avoids the statistical power issues associated with interaction modeling, its scope is limited to a focus on the differential effect between two specific comparisons and may overlook the broader context of how each factor (sex and disease status) independently and jointly affects the outcome. It also includes the implicit assumption that the effects of interest are linear and additive, which may not always be true in biological systems where interactions may be complex and nonlinear.

**Figure 4:**
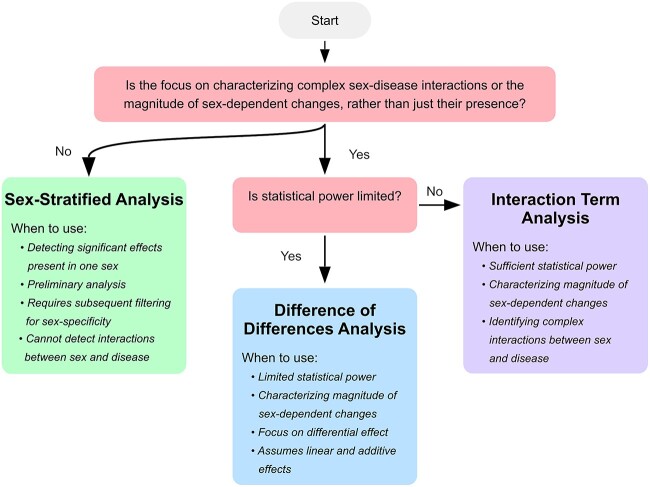
Decision tree for selecting statistical methods to analyze sex-dependent changes in complex diseases. This flowchart guides researchers in choosing between sex-stratified analysis, difference of differences analysis, and interaction term analysis according to the study’s focus and available statistical power. We note that these methods can also be used in combination for a more comprehensive analysis.

In addition to these limitations of different analytical approaches, statistical methods for identifying sex-specific disease-associated changes must always balance the risk of false-positive and false-negative results. Therefore, to increase reliability and comprehensiveness, the use of multi-stage ranking and filtering strategies that incorporate different statistical methods and prior knowledge from the literature is recommended. Such integrated approaches can prioritize the validation of the most robust and biologically plausible sex-dependent molecular changes, i.e. those sex differences that influence not only the magnitude of a change, but also the directionality or presence/absence of an effect, and that are mechanistically explainable, e.g. by known hormonal regulatory patterns affecting disease-associated pathways. Complementary analyses of interactions and differences between sex and disease status can then be used to identify other potential candidates of lower confidence, e.g. changes with sex differences only in magnitude of effect, which can serve as secondary priorities for subsequent validation studies. Figure 4 provides a decision tree to help guide researchers in selecting the most appropriate statistical methods for analyzing sex-dependent changes in complex diseases, depending on the focus of the study and available statistical power.

Overall, univariate statistical methods are particularly well suited to data sets with well-defined variables and relatively straightforward relationships, e.g. clinical trial data, epidemiological studies, and basic molecular profiling data, especially in diseases with well-established biomarkers or clear phenotypic differences between the sexes. However, they may be less effective in capturing the complex, non-linear relationships often present in high-dimensional omics data or in diseases with subtle, multifaceted sex differences.

## Multivariate statistical and machine learning analysis of sex differences in complex diseases

Multivariate analyses can provide significant advances over traditional univariate approaches in the study of sex differences in complex diseases. While univariate analyses examine a single variable at a time, multivariate approaches can analyze multiple variables simultaneously to reveal relationships and interactions that would remain hidden if variables were considered in isolation. This more integrated perspective can be essential for understanding the complex molecular interactions that cause diseases to manifest and progress differently between the sexes, and provides a more comprehensive view of the combinatorial relationships between the biological and environmental factors at play. However, the complexity of multivariate models can also be a drawback, as they often require more statistical power than univariate approaches, are more computationally intensive, and tend to be more difficult to interpret [44]. In addition, the richness of insights provided by multivariate analyses comes with the challenge of distinguishing meaningful patterns from noise, especially in datasets where the number of variables greatly exceeds the number of observations (associated with analytical challenges known in the literature as the ‘curse of dimensionality’ [45, 46]). The influence of confounding factors, bias, and outliers can further complicate multivariate analyses [47–49]. Despite these challenges, the practical advantages of multivariate approaches in revealing subtle, sex-specific disease mechanisms often outweigh their drawbacks in biomedical data analysis.

### Multivariate statistical analysis

Multivariate statistical analysis involves the simultaneous examination of multiple variables to determine the relationships and interactions among them. In the context of sex differences in complex diseases, these analyses can identify patterns and associations that are not apparent when variables are considered in isolation. For example, multivariate regression models can adjust for confounding variables and identify sex-specific risk factors or outcomes. Factor analysis and cluster analysis are other multivariate techniques that group variables or individuals based on underlying patterns, potentially revealing sex-specific clusters of disease presentation or progression.

An example of a software tool in this category is MetaFun, developed by Malmierca-Merlo et al. [50]. This software was designed for statistical meta-analysis of multiple omics datasets to aid in understanding sex differences in diseases of interest, combining different datasets to increase statistical power. Its approach is exemplary of how multivariate statistical analyses can uncover complex interactions and patterns in data that single-variable analyses may miss, providing a more detailed understanding of sex differences in disease mechanisms and progression.

An illustrative applied study is that of Orozco et al. [51], who examined sex differences in effect size estimates at established genetic loci for seven complex diseases using data from the Wellcome Trust Case Control Consortium. By calculating per-allele odds ratios for each sex and relative odds ratios (RORs) of single nucleotide polymorphisms (SNPs), and then synthesizing RORs across multiple loci and different diseases using meta-analysis, Orozco et al. demonstrated the utility of integrating associations across multiple diseases and genetic loci in discerning the dynamics of sex differences in genetic associations.

Another exemplary approach for the sex-dependent analysis of significant SNPs from GWAS data for common diseases was presented by Liu et al. [52]. By using a permutation method to assess sex effects (PMASE) in addition to logistic regression and Woolf’s test for heterogeneity, they identified significant associations between SNPs and diseases such as coronary heart disease and Crohn’s disease that manifest differently between the sexes. Their research underscores the complex interplay between genetic variants and sex and illustrates the utility of multivariate analysis in revealing sex-specific risk factors and outcomes. This study also highlights the benefits of integrating sex-specific considerations into genetic studies, paving the way for more personalized and effective diagnostic and therapeutic strategies that take into account sex differences in genetic predisposition and disease progression.

Moving from individual genetic studies to cross-study integrative analysis, Magi et al. proposed a comprehensive framework for conducting sex-differentiated meta-analyses of GWAS data [53]. They introduced a methodology designed to assess heterogeneity in allelic effects between males and females, allowing the detection of sex-specific genetic associations that may be missed by traditional analyses. Through detailed simulations, their study demonstrated the potential of sex-differentiated meta-analyses to increase the power of GWAS results. The study highlights the utility of such analyses to facilitate the design of more tailored medical interventions and reinforces the value of considering sex as a fundamental variable in the genetic study of disease.

Taken together, the above studies further underscore the importance of incorporating sex-specific analyses into research and highlight the potential of such analyses to enrich our understanding of basic biology and complex disease mechanisms.

### Machine learning analysis

Machine learning provides a flexible approach to analyzing complex datasets, capable of modeling nonlinear relationships and interactions without requiring an explicit specification. In the study of sex differences, supervised learning algorithms such as tree-based predictors (e.g. XGBOOST [54], Random Forests [55], and Optimal Decision Trees [56]), artificial neural networks (e.g. classical feed-forward neural networks, and deep learning networks for datasets of appropriate size and structure [57]), and kernel-based predictors (e.g. Support Vector Machines, Gaussian processes, and Radial Basis Function Networks [58]) can classify individuals into disease categories based on sex-specific patterns in the data. Unsupervised learning, including clustering methods and bi-clustering [59, 60], can discover hidden structures in the data and provide new insights into how disease subtypes may differ by sex.

An exemplary study is the recent work by Johnson and Krishnan on combining public transcriptome data with machine learning to infer age- and sex-specific molecular phenomena across different tissues in the human body [61]. The authors present a computational framework that applies machine learning to analyze large amounts of publicly available human transcriptome data to infer molecular signatures specific to different sex and age groups. Using logistic regression classifiers with elastic net regularization, the study both discriminates between age groups and captures sex-stratified gene signatures in a biologically meaningful manner. These results highlight the utility of machine learning in uncovering sex-dependent molecular characteristics in high-dimensional omics data, and provide a baseline reference for comparing sex differences in complex diseases with those observed in healthy individuals.

For specific human diseases, initial studies have also shown that taking sex-related patterns into account can increase the diagnostic yield of machine learning-based omics analyses. For example, a study on the bioinformatic analysis of AD diagnostic biomarkers in peripheral blood used support vector machines to find discriminative molecular signatures with a focus on sex differences [62]. Building on the prior knowledge that sex significantly influences the manifestations of AD, the study results contribute to a detailed understanding of sex-dependent changes in AD and highlight the utility of sex-specific biomarkers in improving diagnostic accuracy.

Sex differences have also been described for common cardiovascular diseases, and a recent study by Kwak et al. used machine learning to explore sex-specific associations between cardiovascular risk factors and atherosclerotic cardiovascular disease (ASCVD) [63]. The authors developed a random forest model to predict the 10-year risk of ASCVD in each sex, and the association between cardiovascular risk factors and the probability of ASCVD was examined using partial dependency plots. The study revealed multiple sex-specific associations and confirms the importance of considering sex differences in disease-associated molecular data analysis.

Finally, in infectious disease research, sex-specific biomarkers have recently been investigated in molecular data from COVID-19 patients who had different physiological responses to infection [64]. Using a genetic algorithm to explore the space of possible feature subset selections in combination with two machine learning algorithms, support vector machines and logistic regression, both algorithms identified kynurenine and hemoglobin as the most important variables to distinguish between COVID-19-associated changes in males and females. The study highlights the importance of sex differences in the clinical manifestations of the disease and provides insight into potential avenues for personalized treatment strategies.

Collectively, these studies highlight the critical role of sex differences in the analysis of omics data for complex pathologies and demonstrate how advanced machine learning techniques can derive sex-specific molecular signatures with the potential to improve the performance of diagnostic or prognostic models, and identify novel disease-relevant and sex-dependent alterations in cellular pathways. They illustrate that multivariate machine learning approaches are particularly well suited for analyzing large, complex datasets with potentially non-linear relationships, making them valuable for high-dimensional omics data and for uncovering subtle patterns in diseases with complex sex differences. These methodologies can be particularly effective in predictive modeling of disease risk, progression, or response to treatment based on sex-specific factors. However, their ‘black box’ nature can make interpretation challenging, potentially limiting their applicability in clinical settings where clear explanations of findings are critical.

### Dimension reduction methods

**Figure 5:**
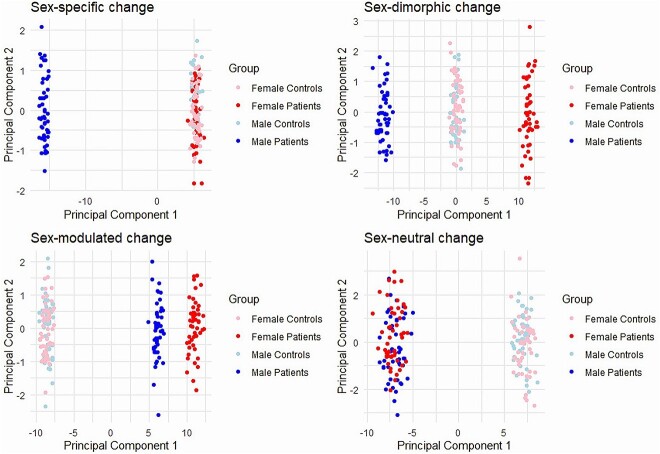
Representative principal coordinate analysis (PCoA) plots for different patterns of sex dependencies in disease-associated molecular abundance changes (using simulated data; the patterns are idealized for illustrative purposes, whereas in real data sets mixtures of different patterns as well as stronger influences of noise and biases would be expected). Top left: Sex-specific change (here only male patients); top right: Sex-dimorphic change (i.e. divergent changes between female and male patients); lower left: Sexmodulated changes (i.e. deviations of the patient from control data in both sexes, but with significantly stronger deviations in one sex than in the other); bottom right: Sex-neutral changes (i.e. the deviation of patient data from control data is similar for both sexes; see also the corresponding box plot examples in Fig. 3). The intentional simplicity of these PCoA emphasizes the conceptual nature of these patterns, facilitating understanding of fundamental principles in sex-based analyses of complex diseases.

Dimension reduction is a commonly used tool in the preprocessing and analysis of high-dimensional data, such as genetic or molecular datasets, where the number of variables greatly exceeds the number of observations. Methods such as principal component and principal coordinate analysis (PCA and PCoA), t-distributed stochastic neighbor embedding [65], and uniform manifold approximation and projection [66] reduce the dimensionality of the data to more manageable levels while attempting to preserve the most important variance or structure within the data. Although dimension reduction can result in a loss of information if the structure of the data is inherently high-dimensional and should be applied with caution, it can be a helpful step in omics data analysis to improve interpretability and facilitate the identification of meaningful biological patterns. When analyzing sex differences, dimension reduction can help visualize and interpret complex patterns and interactions among variables that are specific to males or females. For example, PCoA can reveal global patterns of genetic or molecular markers that differ in significance for disease risk or progression between the sexes, which may be relevant for personalized medicine applications. Similar to box plot analysis of single biomolecules (see Fig. 3), dimension reduction methods can reveal coordinated patterns of sex-specific, sex-dimorphic, sex-modulated, or sex-neutral changes in the data (see Fig. 5), although in most practical cases a complex mixture of these different patterns is observed.

A representative dimension reduction approach with direct applications in the analysis of disease-associated sex differences in omics data is the multifactor dimensionality reduction (MDR)-Phenomics approach by Mei et al. [67]. Their research introduced a new MDR method that integrates genetic factors with phenotypic variables, such as sex or disease status, to better capture the complexity of genetic heterogeneity. By focusing on the interactions between genes and phenotypes, this method is particularly useful in the context of sex differences, allowing researchers to identify patterns and interactions that may be specific to females or males. This approach addresses the need to account for the inherently high-dimensional structure of omics data while minimizing information loss, thereby improving the interpretability of sex-specific biological patterns in complex diseases.

Another study that made a significant contribution to this field proposed a tensor factorization technique for low-dimensional analysis of longitudinal omics data [68]. This method is adept at uncovering the underlying structures and patterns within noisy, high-dimensional data sets, including those related to sex differences in complex diseases. By applying tensor factorization, the study provides an effective tool for visualizing and interpreting dynamic changes in omics data over time, which can provide valuable information for understanding how diseases progress differently between the sexes. This approach complements the MDR phenomics method by providing a way to handle the temporal aspect of omics data, further enhancing the ability to detect sex-specific biological patterns in disease mechanisms.

Finally, a review by Meng et al. discussed integrative dimension reduction approaches to omics data analysis [69]. It highlighted the importance of combining data from multiple omics sources to gain a comprehensive understanding of complex diseases, with potential applications in the study of sex differences. By using dimension reduction techniques tailored for multi-omics integration, different biological data types can be consolidated to provide a combined view of disease mechanisms that may differ between females and males. This integrative approach can increase the interpretability of complex, high-dimensional omics datasets and is particularly useful for identifying common patterns of change across studies that are sex-dependent.

In summary, although dimension reduction methods always carry the risk of information loss due to data compression, they can provide useful insights into sex differences in complex diseases by enabling simplified visual inspection of patterns in omics data. Dimension reduction techniques are particularly useful for high-dimensional data types such as transcriptomics, proteomics, or metabolomics, where the number of features far exceeds the number of samples. They are well suited for exploratory analyses in complex diseases where sex differences may be manifested in multiple, interrelated variables. However, care must be taken when interpreting the results, as important sex-specific signals may be lost in the reduction process.

## Cellular process and pathway analysis of sex differences in complex diseases

While classical statistical analysis and machine learning can provide valuable insights into sex-specific associations and patterns within large datasets, they often fall short of capturing the biological context and complex interactions that underlie sex-specific disease mechanisms. This limitation highlights the need for more structured and knowledge-driven analysis approaches that incorporate prior information from curated cellular process and pathway definitions into the analysis.

Pathway analysis of omics data can be approached through various analytical frameworks, notably over-representation analysis (ORA) or rank-based approaches, and self-contained or competitive statistical tests. ORA compares the frequency of occurrence of differentially abundant biomolecules within predefined pathways to a background list of all measured biomolecules, and identifies pathways with significantly enriched changes in their biomolecule members [70]. In contrast, rank-based approaches, such as Gene Set Enrichment Analysis [71], evaluate the distribution of genes/biomolecules within a ranked list based on their abundance changes, allowing the identification of pathways with coordinated abundance changes without pre-defined significance thresholds. Furthermore, pathway analysis methods can be categorized into self-contained statistical tests, which assess whether genes/biomolecules in a pathway are associated with the outcome of interest independently of other biomolecules, and competitive tests, which assess whether biomolecules in a pathway are more associated with the outcome than those not in the pathway. When applied to sex-dependent pathway analysis in omics data, these methods enable the identification of distinct biological pathways that are differentially affected by sex in a disease state, either by highlighting pathways with a significant concentration of sex-dependent differentially abundant biomolecules (ORA, self-contained tests) or by detecting many subtle but coordinated molecular activity shifts within pathways across sexes (rank-based, competitive tests).

**Figure 6:**
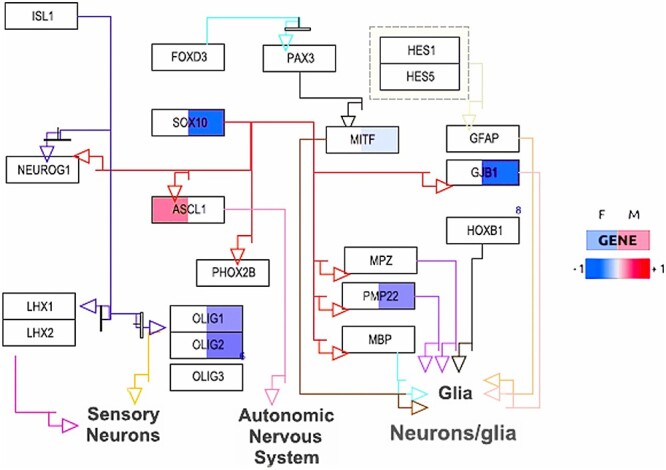
Illustration of sex-specific differential gene expression in the ‘Neural Crest Differentiation’ pathway from the WikiPathways database in Alzheimer’s disease prefrontal cortex RNA-seq data (shown is a segment of the adapted pathway diagram enriched in male-specific changes). Each box represents a gene, and the colors reflect the estimated log fold change in patients versus controls for females (left side) and males (right side). Over- and under-expression is indicated by a blue to red color gradient (see legend on the right), lack of significant expression changes is indicated by a white color. The diagram shows a male-specific overrepresentation of underexpressed genes in patients (in female patients either no significant change in expression is observed or an increased expression, as for the gene ASCL1).

Just as the statistical analysis of individual biomolecules allows the identification of different sex-dependent patterns, such as sex-specific, sex-dimorphic and sex-modulated alterations, pathway analyses can similarly distinguish these patterns by analyzing the enrichment of these categories of biomolecules in known cellular processes. This approach allows the identification of pathways that are uniquely altered in one sex (see example in Fig. 6), show opposite changes between the sexes, or show different levels of alteration between the sexes. However, many pathways may show a complex mixture of these different sex-dependent alteration patterns (e.g. see Fig. 3 in our previous publication on transcriptomic sex differences in Parkinson’s disease [72]), making it difficult to strictly categorize them into one type. Therefore, an unbiased analysis that tests for enrichment of all possible types of variation patterns is recommended to obtain more comprehensive results. This approach ensures that different types of subtle but potentially relevant sex-dependent differences in pathway activities are not overlooked.

Because of this complexity of different sex-dependent patterns occurring within the same pathways, the challenge in pathway analysis of sex differences lies not only in the statistical identification of relevant patterns, but also in their mechanistic interpretation. This requires consideration of the biological context and, typically, manual visual inspection of complex pathway diagrams that integrate different types of alterations. These visual representations help to elucidate how different sex-dependent patterns converge or diverge within specific pathways and how they affect disease mechanisms.

A representative study addressing these pathway analysis challenges was presented by Zhu et al. who performed a systems-level bioinformatics analysis of gene expression profiles to investigate sex differences in ischemic stroke [73]. Highlighting the aspect of interconnected changes in the data, their approach analyzed sex-dependent differential expression not only at the level of individual genes, but also at the level of global pathway changes using statistical enrichment analysis. This facilitated the identification of sex-specific changes in biological processes and provided a richer, contextual understanding of how ischemic stroke manifests differently in the two sexes. The work exemplifies how combining conventional data analysis with knowledge-driven pathway exploration can reveal the biological underpinnings of sex-specific disease mechanisms.

More recently, pathway analyses have been extended to take into account the topology of molecular networks, such as protein–protein interaction (PPI) networks, as illustrated in the study by Lv et al. that also investigated sex-specific modulatory aspects in ischemic stroke [74]. Their work both confirmed the presence of sex differences at the gene expression level and provided a deeper understanding of how these differences influence disease mechanisms through coordinated changes in PPI networks in females and males. By examining gene interactions in the context of known biological pathways and network topology, they provided new insights into the complex, sex-specific modulation of ischemic stroke risk and progression.

Finally, pathway analysis can also provide a useful tool for comparing sex differences between different diseases. For example, Yu et al. explored potential similarities in sex-dependent changes for key genes and pathways between COVID-19 and chronic kidney disease [75]. This research broadens the application of pathway analysis in the study of sex differences by comparing gene expression and pathway regulation in different human diseases, thereby providing insights into common and unique sex-specific mechanisms. Pathway-based investigations are particularly suited for cross-disease comparisons because, while the overlap of individual gene alterations between different diseases is often limited, shared susceptibility mechanisms often become apparent when examining the convergence of alteration patterns at the level of global changes in pathway activity.

In summary, although pathway analyses require high-quality curated information on the cellular processes and signaling pathways of interest, they can provide a more global view of coordinated molecular changes and more interpretable insights into the complex interplay of sex-dependent molecular changes in disease mechanisms. These methods are well suited to provide a biological context for observed sex differences, especially in omics studies where thousands of molecules are measured simultaneously. They are especially useful in diseases where sex differences are thought to operate through specific biological pathways or processes. However, their effectiveness is limited by the completeness and accuracy of existing pathway knowledge, which may not fully capture sex-specific biological processes.

## Mechanistic analysis of disease-associated sex differences in molecular signaling and regulatory networks

The study of molecular signaling and regulatory networks to understand sex differences in complex diseases, as opposed to traditional pathway analysis methods, is motivated by the need to more comprehensively capture the full spectrum of dynamic interactions in biological systems. While pathway analysis provides valuable insights into the static relationships between biomolecules within manually curated, predefined pathways, it may not fully account for the intricate, context-dependent interactions and feedback mechanisms that characterize cellular processes. Signaling and regulatory network analysis can provide a more detailed view by mapping the complex web of interactions that govern cellular behavior, including transient interactions and regulatory loops that may significantly impact disease pathogenesis in a sex-specific manner.

**Figure 7:**
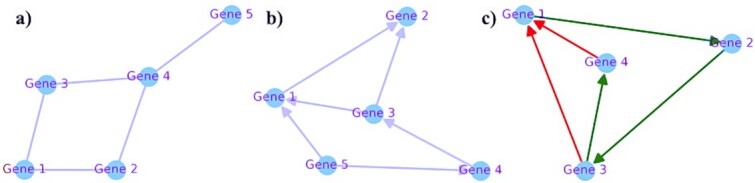
Illustration of three types of network representations used in biological network analysis: Undirected network graph (e.g. used for protein–protein interaction network analysis); b) directed network graph (e.g. used for signaling network analysis of phosphorylation chains); c) directed network graph with activating interactions (e.g., from gene 1 to gene 2) and inhibiting interactions (e.g., from gene 3 and gene 4 to gene 1; used for transcriptional regulatory network analysis of activating and repressing transcription factor (TF)-target relationships). While regulatory and signaling network analyses take into account information about the topology, directionality, and nature of molecular interactions, most conventional pathway analyses do not fully incorporate this information.

**Figure 8:**
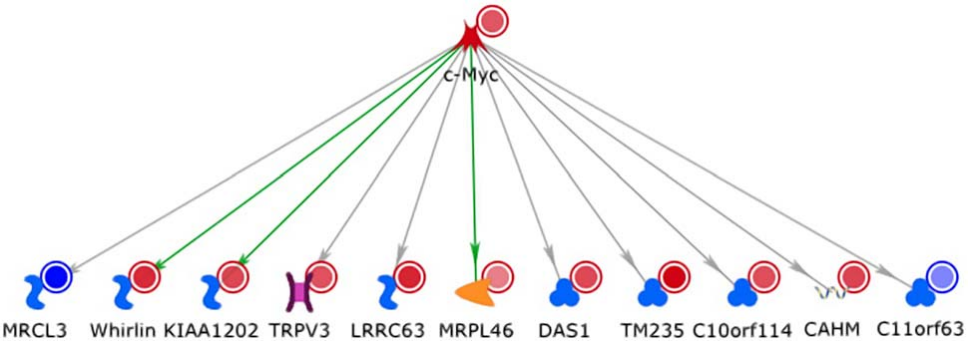
Example of a regulatory network containing genes with sex-modulated expression in Alzheimer’s disease (AD) prefrontal cortex RNA-seq data. Each network node corresponds to a gene, and the colored circles next to the gene represent the log fold changes in a linear model analysis comparing the expression difference between female cases and controls to the difference between male cases and controls. Blue colors represent genes with a significantly lower fold change in AD versus control expression in females than in males, and red colors represent genes with a significantly higher fold change in AD versus control expression in females than in males. The direction of the arrows indicates the regulatory pattern, highlighting that the transcription factor c-Myc controls the expression of the other genes in the graph (green arrows represent activating interactions, gray arrows correspond to interactions with unknown downstream effect; network data were retrieved from human interactions in the mammalian ResNet database using GeneGO MetaCore software, version Q1–2024). Increased expression of c-Myc in neurons in neurodegenerative diseases has been reported to lead to neuronal cell death and the subsequent development of a neurodegenerative phenotype [76, 77].

More importantly, not all molecular interrelationships are captured in known, expert-curated pathways. While existing pathway databases are invaluable, they represent a compilation of only the best studied and documented interactions, whereas genome-scale molecular networks constructed from high-throughput experimental data, such as yeast two-hybrid screens, can provide a more unbiased and comprehensive view of molecular interactions. Furthermore, regulatory and signaling analyses of molecular networks, in contrast to most conventional pathway analyses, are characterized by their ability to capture information about the directionality and type of molecular interactions (activating or inhibiting, see Fig. 7 comparing different network representations and Fig. 8 showing an example of a directed regulatory network with sex-modulated expression in AD). This is essential for understanding causal molecular relationships and the mechanisms underlying pathological and protective processes in complex diseases, including how they vary between the sexes.

For the analysis of molecular sex differences, network analyses can also exploit prior knowledge resources on hormone signaling cascades and their regulatory mechanisms. For example, the *Hmrbase* database provides a comprehensive resource of available information on hormones and their receptors [78]. Similarly, the *EndoNet* database captures valuable information about the components of endocrine networks and their interrelationships [79]. By linking omics data on the sex-specific alterations in complex diseases to these existing knowledge resources, the data can be associated with known hormone signaling mechanisms to derive interpretable and biologically plausible explanations for the observed sex-dependent changes.

A representative example of leveraging gene regulatory network analysis to investigate sex differences in complex diseases is the study by Lopes-Ramos et al. which identified sex differences in drug metabolism in colon cancer [80]. This study inferred patient-specific gene regulatory networks to determine how these networks differ between males and females and affect drug metabolism and treatment efficacy in colon cancer. By using an unbiased transcriptome-scale approach, this work revealed novel differential patterns of transcriptional regulation between females and males that might be missed by traditional pathway analysis. The results highlight the importance of dynamic, context-dependent network analyses, independent of static pathway representations, to elucidate sex-specific regulatory mechanisms and provide potential new insights into personalized therapeutic strategies.

Network analysis can also be an effective means to integrate information across multiple omics data types, as demonstrated in a study by Cheng et al. [81]. They presented a network-based multimodal omics analysis framework to identify and characterize immunometabolic endophenotypes underlying sex differences in Alzheimer’s disease (AD). By mapping the detailed subnetworks of relevant molecular interactions and regulatory mechanisms, their study provided new information on coordinated, sex-specific shifts in molecular activity in AD, highlighting the benefits of integrating complementary omics data for a more comprehensive understanding of disease biology.

While most studies on omics network analysis of disease-associated sex differences focus on gene regulatory or protein interaction networks, initial research on disease-relevant sex-dependent patterns in metabolite networks has also been presented. One such study by Li et al. aimed to facilitate the characterization of the diversity in metabolite networks between males and females by investigating the effect of sex on a complex interlinked network constructed from central biochemical metabolites [82]. Although this study focused on the analysis of blood metabolic markers from healthy individuals rather than patients, the topological analysis of a generic differential network between the sexes highlighted several metabolites with high network centrality that may help to explain different responses to disease and stress between the sexes.

As the topology and activity patterns in networks can change dynamically over time, recent studies have also investigated sex-specific network changes in time series data for complex diseases. Among these, the study by Sun et al. stands out as a comprehensive investigation of transcriptomic sex differences in AD, integrating global gene expression profiles and network analyses across different cortical regions and disease stages [83]. Their integrative network analysis revealed that molecular networks are more conserved in females than in males across different cortical regions and stages of AD, and uncovered genes and pathways, such as glycogen synthase kinase 3β, that are only associated with AD clinical features in males. This joint examination of temporal and regional patterns revealed that changes in cellular processes in AD not only exhibit sex-specific profiles, but also significant time and region-dependent variations, illustrating the dynamic nature of disease progression.

Overall, while unbiased network analyses may not provide the same level of interpretability as analyses using manually curated pathway diagrams, they offer a more complete and dynamic view of the molecular mechanisms underlying sex differences in diseases. These approaches can account for the full range of known molecular interactions, including their directionality and regulatory effects, which are essential for delineating causal mechanisms. Such comprehensive analyses can uncover sex-specific network alterations that are instrumental for a better understanding of disease pathogenesis and progression, and for identifying potential therapeutic targets for sex-dependent disease mechanisms. Network analysis approaches are particularly valuable for studying complex diseases where sex differences are thought to arise from system-level properties rather than individual molecules. They are also well suited for integrating multiple types of data and uncovering sex-specific regulatory mechanisms or signaling cascades. However, these methods can be computationally intensive and may require large sample sizes to reliably infer sex-specific network differences.

## Limitations and challenges in analyzing sex differences

The methods described in this review can offer versatile and informative tools for investigating sex differences in complex diseases. However, it is important to acknowledge their specific limitations and the overall challenges in this field of study.

1. Statistical Methods:
  - These methods can be sensitive to outliers and may not capture complex, non-linear relationships in the data.
  - There is a need to balance the risk of false positives (Type I errors) and false negatives (Type II errors) when adjusting for multiple comparisons.
2. Machine Learning:
  - These approaches often require large sample sizes for robust results, especially when dealing with high-dimensional data.
  - There is a risk of overfitting, particularly when sample sizes are small relative to the number of features.
  - Ensuring interpretability of complex models can be difficult, particularly in clinical contexts.
3. Dimension Reduction:
  - These techniques may lead to potential loss of important information during data compression.
  - Selecting the optimal number of dimensions to retain without losing critical sex-specific signals remains a complex task.
4. Cellular Process and Pathway Analysis:
  - These analyses depend on current knowledge of biological pathways, which may be incomplete or subjectively biased.
  - The integration of sex-specific pathway information is often limited by available data in public repositories.
5. Mechanistic Network Analysis:
  - These methods can be computationally intensive and complex to interpret.
  - Validating predicted network interactions, especially those that are sex-specific, presents significant difficulties.

In addition to the limitations associated with specific methodologies, the analysis of sex differences in complex diseases faces several overarching challenges that span across various analytical approaches and impact the field as a whole, including:

1. Data availability: Many existing datasets lack a balanced sex representation or the number of samples per combination of sex and condition is insufficient.
2. Confounding factors: It can be difficult to distinguish true sex-based differences from those due to societal, environmental, or other biological factors.
3. Temporal dynamics: Capturing sex differences that may vary across the stages of disease progression or treatment requires longitudinal studies.
4. Intersectionality: Considering how sex interacts with other factors such as age, ethnicity, and comorbidities increases the complexity of the data interpretation.
5. Reproducibility: Ensuring findings are robust and replicable across different populations and study designs is an ongoing concern.
6. Translation to clinical practice: Bridging the gap between identifying sex differences and implementing sex-specific interventions remains a significant challenge.

Addressing these limitations and challenges requires interdisciplinary collaboration, improved study designs, and continued development of analytical methods tailored to sex-based analyses.

## Conclusions

The detection and analysis of sex differences in complex diseases has far-reaching implications for biomedical research, clinical practice, and public health. By applying analytical methods to interpret sex-dependent alterations, such as those discussed in this review, researchers may advance our understanding of disease mechanisms, improve diagnostic accuracy, develop personalized treatment strategies, refine clinical trial design, and inform public health policy. These approaches provide robust frameworks for quantifying and characterizing sex differences, using machine learning to identify complex patterns, dimension reduction techniques to distill key features, and pathway and network analyses to elucidate sex-specific biological mechanisms. However, to fully realize this potential, several key developments are needed.

First, the integration of diverse data types, including multi-omics and longitudinal data, will provide a more comprehensive view of sex differences across biological scales and throughout disease progression. Second, the development of sex-aware artificial intelligence systems will help to ensure accurate and equitable healthcare applications. Third, the establishment of standardized best practices for sex-specific analyses will improve reproducibility and facilitate meta-analyses across studies.

As the field progresses, it will be essential to move beyond simply identifying sex differences to understanding their clinical implications. This transition will require close collaboration between computational biologists, clinicians, and public health experts. By bridging the gap between analytical insights and clinical practice, we can work toward a more personalized medicine that takes into account the complex interplay between sex and other biological and environmental factors in disease.

Ultimately, the goal of studying sex differences is not to treat men and women as binary, homogeneous groups, but to use sex as a lens through which we can better understand individual variability in health and disease. As we refine our analytical tools and expand our knowledge, we move closer to a future in which medical interventions are tailored not only to biological sex, but to each individual’s unique physiological profile.

In this review, we have examined a range of generally applicable bioinformatics methods that contribute to these goals, highlighting the utility of standard univariate and multivariate statistical and machine learning approaches, as well as the advantages of specialized dimension reduction methods and higher-level pathway and network analyses for understanding the molecular underpinnings of sex-specific disease manifestations. The example studies discussed, especially those using current mechanistic pathway and network analysis methods, illustrate the significant benefits of integrating molecular measurement data with prior biological knowledge to derive interpretable and biologically plausible information about the mechanisms driving sex-dependent variations in human disease.

Given that there are many diseases that exhibit sex-specific differences in disease risk, phenotypes, and molecular alterations, the previous success stories discussed in this review also suggest that there is still significant untapped potential for bioinformatics techniques to shed light on the complex interplay between sex and disease. By moving beyond the conventional view of sex as a mere confounding factor and examining and interpreting sex-dependent influences on disease mechanisms, a more differentiated assessment of sex-specific effects can be achieved even from the re-analysis of already existing omics datasets. This approach can be instrumental in advancing our understanding of disease mechanisms and their sex dependencies. More importantly, taking into account the strong influences of sex on many disease states also holds great promise for refining diagnostic and therapeutic strategies tailored to the biological specificities of each sex. While many of the resources on prior pathway and network knowledge used in the example studies discussed are disease-specific, most of the bioinformatics approaches reviewed are generically applicable to any disease condition. They can provide a blueprint for future studies to elucidate sex-dependent molecular changes and help ensure that sex differences become a central consideration in biomedical investigations.

Beyond the study of sex differences in complex diseases, the bioinformatics methods discussed in this review have other potential applications. These approaches could be adapted to study further biological variables that influence disease outcomes, such as age, ethnicity, or environmental exposures. In addition, instead of a focus on biomedical applications, the methods could be extended to other areas of research, such as developmental biology, evolutionary studies, or environmental science, where complex, multifaceted differences between groups are of interest. Apart from these applications in other fields, promising avenues for further research on molecular sex differences in human disease include a stronger focus on the dynamic aspects of sex-dependent influences, the development of more interpretable machine learning models, and the integration of complementary omics datasets. As the methods evolve, they may enable more nuanced investigations of individual variability in health and disease, potentially leading to improvements in fields such as personalized nutrition or adaptive clinical trial design.

In addition, studies of the interactions between the variable sex and other disease-modulating and confounding factors are needed to better understand how sex influences disease risk and progression in the context of geographic background, environmental exposures, and other factors. Ultimately, by linking the general investigation of molecular sex differences with more tailored mechanistic studies of sex-specific treatment responses, this research has the potential to provide more effective, personalized medical interventions for both sexes.

**Key Points**

- Many complex diseases exhibit pronounced sex differences that affect disease risk, progression, and response to treatment. Systematic exploration of sex-specific disease mechanisms can significantly improve our understanding of pathological and protective processes with sex-dependent profiles.
- Dedicated bioinformatics approaches are needed to study molecular sex differences in complex diseases, integrating molecular datasets with existing knowledge of hormone signaling, gene regulatory networks, and sex-linked genes. Rather than considering sex as a confounding variable, such dedicated integrative analyses allow for a more complete and mechanistic interpretation of sex-related changes.
- Pathway and network analysis techniques are essential tools for interpreting sex-specific, sex-dimorphic, and sex-modulated changes in diseases at the level of cellular process activities. These analyses can lead to the discovery of more robust sex-specific biomarker signatures and candidate therapeutic targets, and provide new insights into the regulatory and signaling mechanisms underlying disease-associated sex differences.

## Data Availability

The data used in this project was generated by simulation in R (see ‘Code availability’ section and the GitLab repository: https://gitlab.lcsb.uni.lu/bds/sex-differences-review).

## Code availability

The analyses to generate simulated data and associated figures were implemented in R (v4.2.0), and the code is available under the MIT license in the following GitLab repository: https://gitlab.lcsb.uni.lu/bds/sex-differences-review.
